# Returning an extirpated native carnivore to its former range: a habitat suitability analysis of Kangaroo Island for spotted-tailed quolls (*Dasyurus maculatus*)

**DOI:** 10.1038/s41598-026-56577-4

**Published:** 2026-09-15

**Authors:** Mia Retallack, Bertram Ostendorf, Bronwyn Fancourt, David Peacock

**Affiliations:** 1 School of Biological Sciences, Adelaide University, Adelaide, SA 5000 Australia; 2 School of Environmental and Rural Science, University of New England, Armidale, NSW 2351 Australia; 3 Queensland Parks and Wildlife Service, Department of the Environment, Tourism, Science and Innovation, Toowoomba, QLD 4350 Australia; 4 School of Animal and Veterinary Sciences, Adelaide University, Roseworthy, SA 5371 Australia

**Keywords:** Ecology, Ecology, Zoology

## Abstract

Native predators have declined significantly in range and abundance globally. In Australia, the spotted-tailed quoll’s (*Dasyurus maculatus*) range has contracted by up to 30% since Europeans arrived in the late 1700s, with its extirpation from Kangaroo Island sometime after 1837. A feral cat eradication program is currently underway on Kangaroo Island which, if successful, will leave a vacant predator niche. The spotted-tailed quoll has been proposed as the most suitable species to reoccupy this niche, however following its local extirpation, over 50% of the island’s vegetation was cleared, mostly during the 1940s–50s, resulting in uncertainty about the current habitat suitability for spotted-tailed quolls. We used MaxEnt and fuzzy modelling to determine if Kangaroo Island in its current state is suitable for reintroduction of the spotted-tailed quoll. MaxEnt modelling suggested that mean annual rainfall and temperature, and soil clay and sand percentage have the greatest influence on habitat suitability. Fuzzy modelling identified distinct patterns of potential den site availability, with > 50% of Kangaroo Island providing habitat containing a suitably large proportion of den sites. Cape Gantheaume, south and south-western Dudley Peninsula and large areas of western Kangaroo Island support the highest habitat suitability and could be suitable initial reintroduction sites.

**Supplementary Information:**

The online version contains supplementary material available at https://doi.org/10.1038/s41598-026-56577-4.

## Introduction

The extinction of native species is of global concern^1^. Human induced habitat loss, the introduction of non-native species and pathogens, persecution, and climate change are all contributing to the increase in extinction rates^1,2^. Native species, which have not evolved alongside introduced species, often do not have the required mechanisms to deal with the impacts of introduced species, including predation, excessive herbivory, hybridisation, competition for resources, disease transmission, and habitat alteration. These threats make native species particularly vulnerable^2–4^. The cyclical nature of the interaction between these threats and the species that they affect results in the loss of ecosystem function over time, and sometimes, ecosystem collapse^5^. This loss and collapse can be improved through the restoration of species. Rewilding involves the reintroduction of species to places from which they were extirpated, and has the potential to restore lost ecological function^6^. In particular the reintroduction of native predators can help to reduce or even reverse damage caused by the introduction of non-native prey species and/or overabundant native species^7,8^.

Approximately 90% of Australia’s native species are endemic, so the loss of these species is of particular concern^9^. At least 29 species of Australian mammal have gone extinct since the arrival of Europeans in Australia, and approximately 18% of Australian mammal species are currently threatened with extinction^9^. The loss of native Australian predators is particularly notable, with all members of the marsupial carnivore guild either extinct or currently threatened with extinction^10^. This leaves ecological niches available for exploitation by introduced predators such as the red fox (*Vulpes vulpes*) and feral cat (*Felis catus*), which have also contributed to creating the vacancies in these niches^11^.

Kangaroo Island is a 4405 km^2^ island located off the coast of South Australia^12^ with relatively similar flora and fauna communities to those found on the nearby mainland^13^. At the time of European arrival in the early 1800s and their official settlement in 1836^14^, Kangaroo Island supported a diverse range of native predators^15^, including Rosenburg’s goanna (*Varanus rosenburgi*), the tiger snake (*Notechis scutatus*), the pygmy copperhead snake (*Austrelaps labialis*), various birds of prey, the Kangaroo Island dunnart (*Sminthopsis griseoventer aitkeni*), the brush-tailed phascogale (*Phascogale tapoatafa*) and the spotted-tailed quoll^16–19^. Most of these species are still extant, however the quoll and phascogale have since been extirpated from the island, sometime after 1837 and 1839 respectively^20,21^, leaving the island with no medium-large native mammalian predator.

**Fig. 1:**
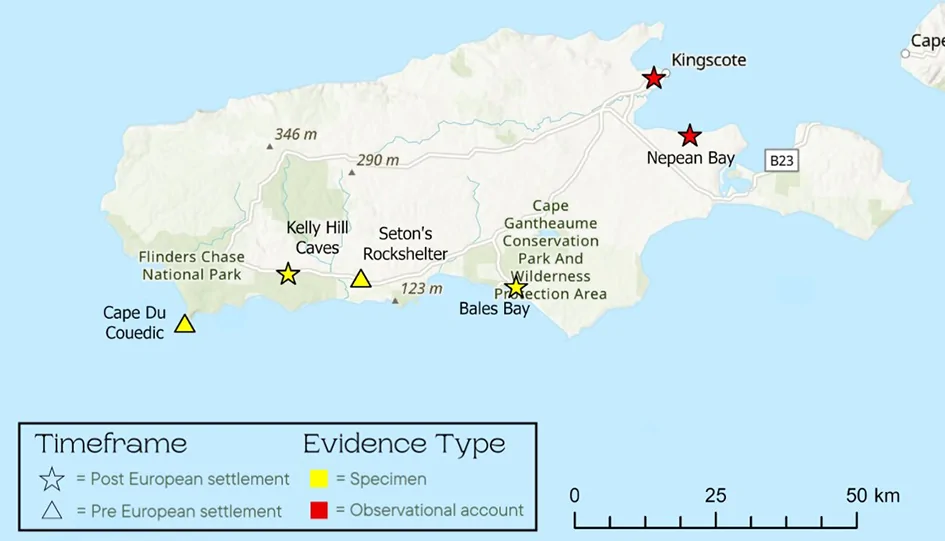
The six locations on Kangaroo Island at which spotted-tailed quoll specimens or observational accounts have been found. Stars represent locations from records that indicate the presence of spotted-tailed quolls on the island after the arrival of Europeans, and triangles represent locations prior to European arrival. The red symbols represent observational accounts while the yellow symbols represent specimens. The Nepean Bay location is proposed based on Nicholas Baudin mooring his ship in this vicinity, but the observation could have been made on another Kangaroo Island shoreline. Created using *ArcGIS Pro Version 3.5.1*^55^. Background sources: Esri, HERE, Garmin, Foursquare, FAO, METI/NASA, USGS.

The spotted-tailed quoll is the only medium-sized native mammalian predator known to have persisted on Kangaroo Island until post-European settlement^15,16^. The spotted-tailed quoll is evident in Kangaroo Island’s fossil record, with specimens between 13,100 and 900 years bp found in Cape Du Couedic, Kelly Hill Caves, and Seton’s Rockshelter^16^, but was, until recently, thought to have disappeared from the island before the arrival of Europeans. However, observational accounts recorded in 1803 by François Péron reported “on the shores … [they saw] the genre of pretty Dasyures”^22^, and in 1837 W. H. Leigh described in great detail his encounter with a spotted-tailed quoll attacking domestic chickens in Kingscote^20^. These accounts suggest that the species persisted on the island for much longer than was previously thought. This hypothesis is further supported by specimens on the island which have been dated to ~ 200 years bp at Kelly Hill Caves^16^, and at Bales Bay at a wallaby skinner’s site^16,23^ (Fig. 1).

The cause of the spotted-tailed quoll’s extirpation from Kangaroo Island is unknown. Observational accounts suggest that the species persisted on the island until at least the late 1830s^20^. Broadscale land clearing on Kangaroo Island did not commence until the early 1900s, with extensive clearing occurring in the late 1940s and early 1950s following the arrival of post-war soldier settlers^24^. The earliest recorded cat on the island was in 1844^25^, and there is no evidence of other introduced predators such as canids (dingoes/dogs and their hybrid *Canis* spp. or red foxes) on the island, hence any potential contribution by introduced predators to the decline of the spotted-tailed quoll on Kangaroo Island is unknown. Human persecution of spotted-tailed quolls is recorded on Kangaroo Island^20^, with Walshe (2014) reporting at least 25 individuals in a wallaby skinner’s bone deposit^23^, suggesting that human persecution potentially contributed to their decline. However, noting the largely intact and densely vegetated habitat from which they were extirpated, hunting by humans would have been difficult, so the possible impact of disease has also been considered^15^. There are numerous records of disease affecting quolls on the Australian mainland^26,27^, however no records of disease affecting spotted-tailed quolls Kangaroo Island have been found^15,26^.

The current absence of medium-large native mammalian predators on Kangaroo Island has exacerbated the abundance of feral cats^28^. Feral cats are implicated in the decline and extinction of many Australian native species, especially on islands^29^. This is of particular concern for native species on Kangaroo Island, some of which are already threatened with extinction^30,31^. For example, the Kangaroo Island dunnart (*Sminthopsis griseoventer aitkeni*) is known to be depredated by feral cats^32^. The impact of feral cats is likely to have been exacerbated by extensive land clearing^33^. Feral cats not only depredate native fauna on the island but also spread the parasite *Toxoplasma gondii* that causes the fatal disease toxoplasmosis. Livestock productivity is also negatively impacted by toxoplasmosis, which together with the cat-borne disease sarcocystosis costs the Kangaroo Island sheep industry in excess of $12 million p.a. through reduced marking rates and increased carcass trimming (B. Fancourt, personal communication, 4 November 2023). The combined impacts of feral cats on both wildlife and agriculture have driven a program to eradicate feral cats from the island, commencing with the Dudley Peninsula in the east. Should this eradication program be successful, the spotted-tailed quoll could fill the vacant predatory niche that will remain. Furthermore, the reintroduction of spotted-tailed quolls to Kangaroo Island will create an additional refuge population, which could act as an insurance population for the species, should populations continue to decline on the mainland.

Given the extensive changes that have occurred on Kangaroo island since European settlement, the present-day suitability of Kangaroo Island for spotted-tailed quolls is unknown. The species’ persistence until European settlement suggests that the island contained habitat for the species at least until that time. However significant habitat clearing has occurred on the island since the spotted-tailed quoll was extirpated. Around 52% of Kangaroo Island now comprises cropland, pasture, and urban environments, with only ~ 48% of the island comprising remnant native vegetation^34^.

**Fig. 2:**
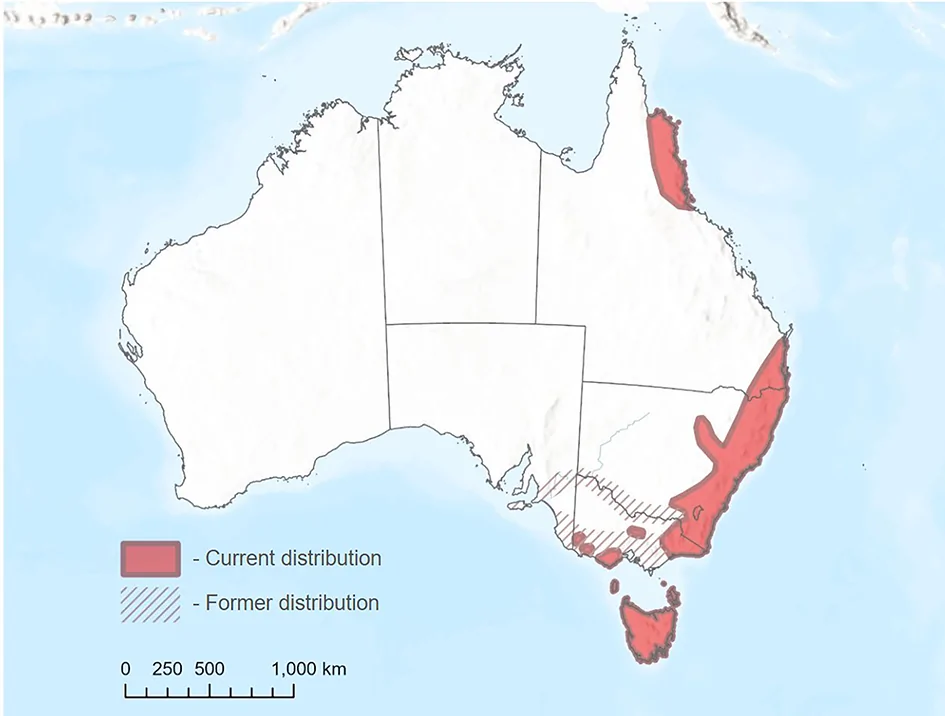
The distribution of the spotted-tailed quoll, including its current and former distribution^88^. Created using *ArcGIS Pro Version 3.5.1*^55^. Background sources: Esri, TomTom, Garmin, FAO, NOAA, USGS, © OpenStreetMap contributors, and the GIS User Community, USGS.

Critical habitat requirements for the spotted-tailed quoll can be inferred from extant populations that persist elsewhere in Australia (Fig. 2). Across their entire range, spotted-tailed quolls prefer productive landscapes with moderate temperature, avoid cleared land (except when travelling), and use linear features such as gullies, fences, and ecotones for movement and foraging^35^. However, there are differences in habitat preferences between Tasmanian and mainland quoll populations. On the mainland, spotted-tailed quolls prefer non-fragmented areas (except where fox density is low^36^, high elevation, high rainfall locations, while in Tasmania they are seen to prefer low elevation areas, with rainfall, topography, forest cover and fragmentation having little effect on their overall distribution^37^. In Tasmania, spotted-tailed quolls are observed to prefer non-eucalypt forests^37^, while eucalypt forests, which tend to have a more complex understory, are preferred on the mainland^38^. Perhaps most interesting is the observed differences in den preferences, with mainland quolls using den sites that are considered more protective such as tree hollows, rocks, and burrows, and have never been reported as using grass or foliage dens^39–42^, while in Tasmania, grass and foliage dens are used preferentially over other types of dens^37^. Overall, these differences suggest that the generally accepted ‘forest dependence’ of spotted-tailed quolls actually reflects the species’ realised niche in areas with introduced canids, but that populations occurring in locations with little or no interaction with canids have more flexible habitat requirements suggestive of their preferred niche^36,37^. Considering the absence of wild canids on Kangaroo Island, it is likely that the habitat requirements for spotted-tailed quolls on Kangaroo Island would be as similarly flexible as they are in Tasmania.

We aimed to assess the current habitat suitability of Kangaroo Island for spotted-tailed quolls, to inform their possible reintroduction. We had three major objectives: (1) determine the most influential environmental variables on the distribution of spotted-tailed quolls and how these variables affect their distribution at a broad scale, (2) use these variables to predict the present-day suitability of Kangaroo Island for the persistence of spotted-tailed quolls, and (3) create a fine-scale habitat suitability map of Kangaroo Island, identifying key areas that could represent suitable reintroduction sites.

## Methods

### MaxEnt (maximum entropy) species distribution modelling

We used MaxEnt modelling at a broad scale to encompass the entire current and historic range of the species, including South Australia, Victoria, New South Wales, Queensland, and Tasmania^47^. We used it to identify the most influential environmental variables on species distribution and to determine the suitability of Kangaroo Island for spotted-tailed quolls relative to the rest of their distribution.

MaxEnt modelling^43^ is a method commonly used in species distribution modelling to predict a species’ range using presence-only data^44^. MaxEnt is a machine learning algorithm that can reflect complex relationships between environmental variables and species occurrence and is thus prone to overfitting due to the biased and/or under-representative nature of species occurrence data^45^. There are six key ways in which the output and interpretation of MaxEnt models can be significantly altered to control detail of the model response: background data, features, regularisation, sampling bias, output types, and model evaluation^46^. We used the Area Under the Curve (AUC) statistic to assess the models^48,49^.

### MaxEnt occurrence data

For MaxEnt modelling, we used species occurrence datasets from The Atlas of Living Australia^50^. The search for ‘***Dasyurus maculatus***’ produced 14,723 records, including records from museums, citizen science websites such as iNaturalist^51^, as well as government and council databases. We only used observations of living animals. We excluded museum locations and occurrence records that were non-living at the time of recording (e.g. specimens and material samples) as these records may include animals that were alive prior to changes in habitat or may have moved over time (e.g. by intervention from other animals, or as a result of weather events). In addition, to mitigate the effects of survey bias^52^ we thinned the data using the spThin package^53^ in Program R V4.3^54^ to remove all occurrence points within 5 km of one another, matching the spatial resolution of the lowest resolution dataset (rainfall). Spatial thinning has been shown to increase model performance and reduce overfitting^52^. Finally, we visually identified and manually removed spatially questionable points, such as those with coordinates placing them in the ocean, using ArcGIS Pro^55^. This resulted in a set of 2,380 records for further analysis. The original thinned and cleaned datasets are included in supplementary information Fig. 1 and supplementary Table 1.

### MaxEnt variable selection

The effect of biophysical variables on the distribution of quolls is reasonably well established^37,56^. Spotted-tailed quolls avoid mid-slopes, but differences in climate and elevation preference occur between mainland and Tasmanian populations^37,56^. These effects may be related to interactions between temperature and terrain. Therefore, rainfall, temperature, elevation, ridgetop flatness, and valley bottom flatness were included. To investigate the effect of soil characteristics on distribution, depth to regolith, soil clay, sand, and silt percentage were also assessed. The datasets used in preliminary analyses were available through the data portal on EcoCommons^57^. To increase parsimony of the model, we initially used the Biodiversity and Climate Change Virtual Laboratory to run preliminary MaxEnt models that included the full set of variables. We removed variables with high correlation (> 70%) and variables with the lowest permutation importance to avoid the inclusion of data that were performing similar functions in the model, or performing little to no function at all^46^. The resulting variables included in the final model were soil clay percentage at a depth of 60–100 cm^58^, soil sand percentage at a depth of 15–30 cm^59^, mean annual rainfall (1991–2020)^60^, and mean annual temperature (1991–2020)^61^, where temperature and rainfall were more influential than edaphic variables. The final variables selected are displayed in supplementary information Table 3. In order to run models with the high resolution and broad scale of the soil raster layers, and to utilise full levels of diagnostics, we ran the final MaxEnt model using the ENMevaluate package^62^, in Program R V4.3^54^.

### MaxEnt model settings

We used the default settings for the selection of background samples, which assumes that the species is equally as likely to occur in any one location compared to another, and therefore the model gives every pixel an equal chance of being selected. We included all feature classes (linear, quadratic, hinge, product, and threshold (LQHPT)), which is most appropriate when there are greater than 80 occurrence points being modelled^46^. Regularisation multiplier (rm) is used to adjust smoothing and complexity of fits with larger occurrence point datasets allowing lower rm values. We selected a value of 0.5 for this model^63^. Sampling bias was avoided by thinning the occurrence data, as described previously, and we used the Cloglog output type as it is generally accepted to be the most appropriate output for estimating probability of species presence^43^.

### Fuzzy logic and its integration with modelling

Fuzzy logic underpins approximate forms of reasoning, and is used in cases where precise reasoning may be limiting^65^. In traditional logic systems, a statement is either true or false, while using fuzzy logic allows for the truth of a statement to be represented across a scale, where a value of one indicates complete truth, and a value of zero indicates a total lack of truth^65^. It has been used in many aquatic habitat suitability models, however few studies have implemented the technique for terrestrial habitat modelling, so this is still a relatively novel approach^63,64^. Fuzzy habitat suitability modelling can interpret vague, poorly understood data, or that for which only qualitative descriptors are available. This makes it suitable for interpreting ecological data, which often falls into these categories. Ecological data is also inherently imprecise, and the use of fuzzy logic in its interpretation helps to mitigate the effects of this imprecision^66,67^.

### Fuzzy modelling variable selection

We performed fuzzy modelling in ArcGIS Pro^55^, using the fuzzy overlay and fuzzy membership tools. We selected variables using a combination of preliminary MaxEnt outputs, discussion with species experts, and through a review of published literature on spotted-tailed quoll habitat use. We selected variables that reflected the fine-scale habitat requirements of spotted-tailed quolls, with a particular focus on denning requirements, as female distribution is largely determined by the availability of resources, particularly den sites and prey availability^39^. The overlap between the diets of feral cats and spotted-tailed quolls^32,68^, and the ability for cats to sustain themselves at high abundance on Kangaroo Island^28^, suggest that prey is sufficient, and therefore den site availability is the most likely significant limiting factor. Male distribution is limited by female distribution and therefore is also indirectly limited by den site availability^39^.

We selected spatial variables to either reflect den site availability, or in accordance with demonstrated climatic and edaphic preferences demonstrated by MaxEnt modelling. We defined a den site as any site which a spotted-tailed quoll may be inclined to use to sleep in or to care for young based on variables defined in the literature^39–42^. There is insufficient available data on the maternal denning requirements of spotted-tailed quolls compared to sleeping dens^37,69^, so we did not distinguish the two types of dens. Maxent modelling demonstrated influence of temperature, rainfall, soil sand percentage, and soil clay percentage on quoll occurrence at a national scale, however we excluded temperature from fine-scale modelling on Kangaroo Island as the entire island falls within a value of mean annual temperature that has been determined by MaxEnt as being suitable for spotted-tailed quolls. We also excluded Soil sand percentage due to a high negative correlation between soil sand percentage and soil clay percentage across Kangaroo Island. The permutation importance of soil sand and clay percentage were similar, and variation across the island was similar, but inverse, so selection of clay over sand was arbitrary, and the use of either variable produced similar results in preliminary testing. To reflect denning suitability and to allow the model to reflect suitability at a finer scale, we included a measure of understory density derived from the National Vegetation Information System (NVIS)^70^, and a LiDAR derived foliage cover model^71^ in the analysis. The variables used in the fuzzy logic models are outlined in supplementary information Table 4.

### Fuzzy membership

For habitat suitability modelling in ArcGIS Pro, we reclassified each variable raster layer to assign each cell a value between zero and one, which described the likelihood of that cell belonging to a suitable class. This was done using the fuzzy membership tool, within which variables can be reclassified according to seven different membership types, outlined in supplementary information Table 5. We used response curves for rainfall and soil clay percentage, which were returned as an output of MaxEnt modelling (supplementary information Figs. 3 and 4), to determine ideal ranges of values for these variables. Response curves indicated that spotted-tailed quolls tend not to inhabit areas with < 450 mm mean annual rainfall, but that there is a steep, almost linear increase in suitability beyond this point, plateauing at approximately 800 mm mean annual rainfall. This is consistent with a published preference for a climate with > 600 mm mean annual rainfall^72^. Therefore, we reclassified rainfall according to the linear fuzzy membership type, and set the minimum to 450 mm, and the maximum to 800 mm. This reclassified the variable such that any cell with a value of ≤ 450 mm was assigned a membership value of zero, and any cell with a value of ≥ 800 mm was assigned a membership value of one. Values between 450 and 800 mm were assigned membership values increasing linearly between zero and one. Response curves also indicated a preference for lower clay content, with an almost linear decrease in suitability between 8% clay content and 55% clay content. Therefore, we used the linear membership function to reclassify the soil clay percentage layer, where 55% was set as the minimum and 8% as the maximum, such that anything with ≤ 8% clay content would be assigned a value of one, and anything with a ≥ 55% clay content would be assigned a value of zero, with a linear decrease in membership between the two values. While the reclassification of variables does not necessarily exactly represent the response of the species to each variable, as the response to each variable is not linear, it acts as an approximation.

**Fig. 3:**
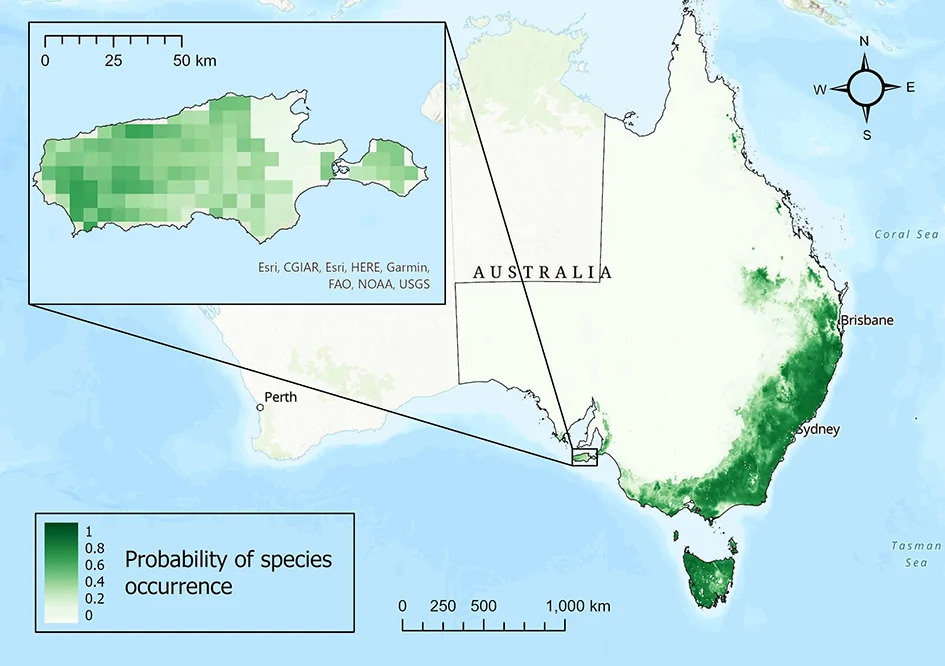
Maxent model of the eastern half of Australia, including a zoomed in map frame of Kangaroo Island. Darker green areas represent a higher habitat suitability and therefore higher probability of species occurrenceCreated using the ENMevaluate package^62^, in *Program R V4.3*^54^ and *ArcGIS Pro Version 3.5.1*^55^. Background sources: Esri, USGS, Garmin, FAO, NOAA.

**Fig. 4:**
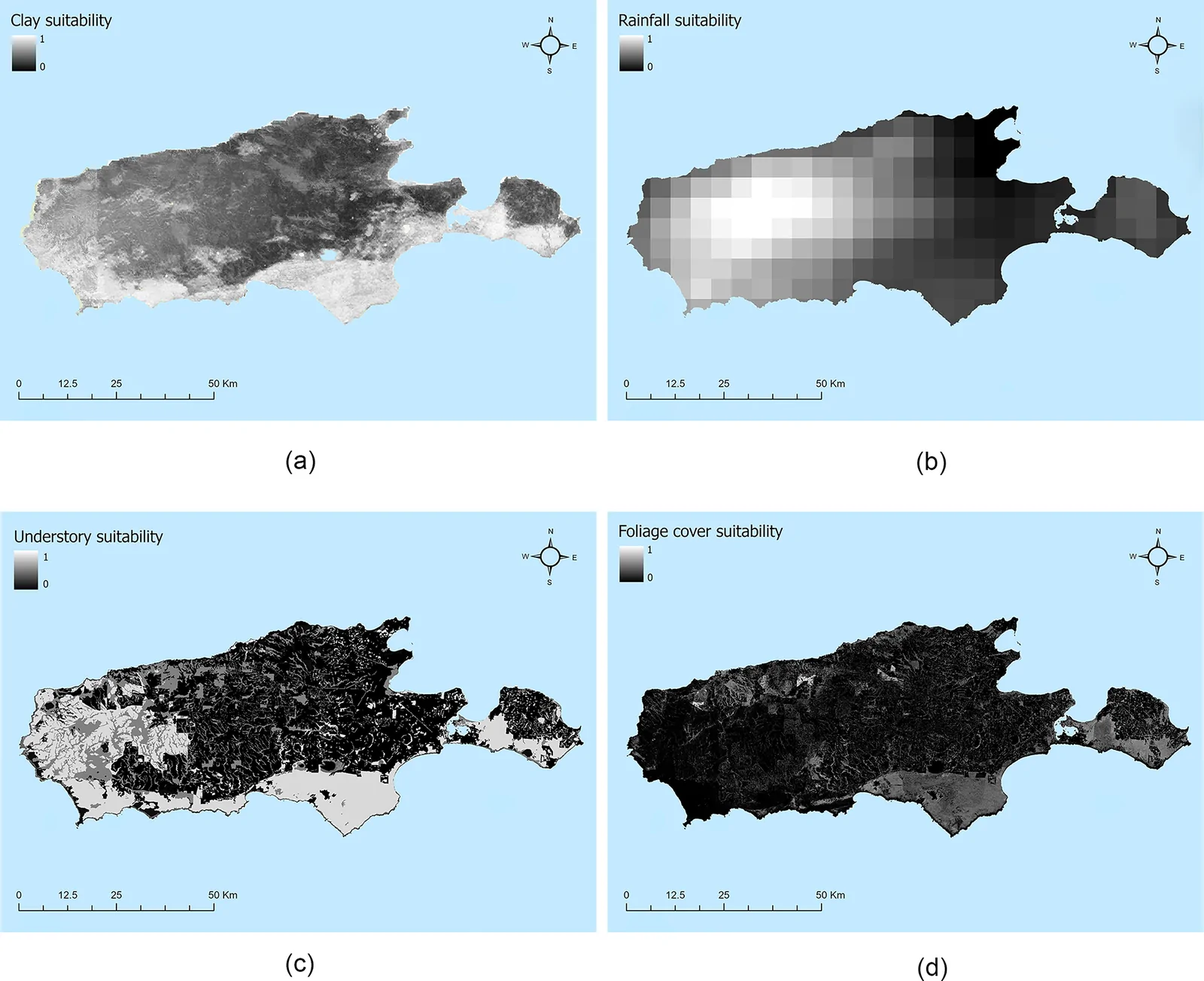
Maps of Kangaroo Island depicting the fuzzy membership layers, representing suitability of soil clay percentage (top left), mean annual rainfall (top right), understory density (bottom left), and foliage cover (bottom right). Created using *ArcGIS Pro Version 3.5.1*^55^

We estimated ideal values for foliage cover and understory vegetation density based on a review of literature and discussion with experts, but as neither variable was included in the MaxEnt modelling due to a lack of a suitable and available Australia-wide dataset, we could not obtain response curves for either variable. Increasing foliage cover may reflect increasing prey availability^73^ and would benefit spotted-tailed quolls by increasing cover from raptors^69^ and aiding in thermoregulation^74^, but may also increase the likelihood of den site occurrence by providing a higher density of leaf litter and branches that may be used as dens^37^. Therefore, we reclassified foliage cover using the fuzzy membership tool with a linear membership type, where we assigned cells with 0% foliage cover a membership value of zero, and cells with 100% foliage cover were assigned a membership value of one, and values in between 0% and 100% were assigned values between zero and one using a linear membership function. Finally, we included understory density as a measure of den site availability. Due to the absence of burrowing species such as rabbits or wombats and the lack of large predators on Kangaroo Island^19,75^, spotted-tailed quolls are likely to utilise grass and foliage dens as they do in Tasmania^37^, which is also free of wild introduced canids. Therefore, increased understory density acts as an indicator of den site availability. Like foliage cover, increased understory density may also contribute to increased numbers of small mammals^76^, increasing prey availability, and may also aid in thermoregulation^74^. Due to the configuration of the NVIS dataset, the fuzzy membership tool could not be used for reclassification of the dataset. Instead, the NVIS structural formation terminology, as defined in Table 7 of the Australian vegetation attribute manual^77^, was used to define the approximate understory density of each major vegetation subgroup (MVS), based on the MVS name. When an understory layer was described in the MVS name, the median value of the percent cover defined by the NVIS structural formation terminology was used to define it. When more than one understory type was defined, the median value of the total maximum and total minimum percent cover was used, and when no understory layer was described but the area was defined as being vegetated, the percent cover was nominally defined as 5% to differentiate these areas from those that were non-vegetated. Freshwater, sea, estuaries, naturally bare, unclassified areas, and polygons with no data were defined as having 0% cover. Percent cover was then transformed into a fuzzy ranking by transforming the percentage value to a proportion. The resulting definitions of percent cover for each vegetation type are shown in supplementary information Table 6. The fuzzy ranking for each MVS category in ArcGIS Pro was manually defined, and the polygon to raster tool was used to transform this layer, based on the fuzzy ranking values, so that it was available for use in the fuzzy overlay tool.

## Fuzzy overlay

Once each raster layer had been reclassified, we input them into the fuzzy overlay tool in ArcGIS Pro^55^. The fuzzy overlay tool combines raster layers according to a user-specified algorithm known as the overlay type, of which there are five, outlined in Appendix E. We selected the fuzzy gamma overlay type for its ability to return a value that is a combination of input values, without returning such extreme values as fuzzy product or fuzzy sum^78^. The choice of the most suitable value of gamma is not based on specified constraints, but rather on the desired output of the model. Lower gamma values tend to produce more spatially constrained results, and it is generally accepted that values near to the default value of 0.9 are suitable^79^. Dias et al.^80^ demonstrated that when using a gamma value of 0.85, the habitat suitability model found an area of approximately 70 km^2^ to be of high or extremely high management priority, while when using a gamma value of 0.95, the area found to be of high or extremely high management priority encompassed an area of approximately 700 km^2^. This suggests an approximately 900% increase in high and extremely high priority management areas, demonstrating the power of alteration of the gamma value. With no reason to select for either a more or less constrained area of suitability, we selected the default gamma value of 0.9 for analysis. We ran fuzzy overlay at a resolution of 10 m to match the finest resolution of the input layers.

### Smoothing

The output of the fuzzy overlay step produces a map that shows the suitability of locations for denning at a resolution of 10 m, essentially depicting the probability of a den site existing at a particular location. However, an individual quoll’s home range does not need to consist entirely of locations suitable for denning. While den sites are important, quolls are able to use open land and ecotones between native vegetation and cleared land for foraging and travel^37^. To assess which areas may be suitable as a home range for spotted-tailed quolls, we created two smoothed models to represent the density of den site availability within the home range of an individual. Firstly, we used the Con tool to reclassify the fuzzy overlay layer into a binary prediction of suitable/unsuitable habitat, giving any cell with a fuzzy value of 0.5 or greater a value of one (suitable), and any cell with a fuzzy value of less than 0.5 a value of zero (unsuitable). We selected this value somewhat arbitrarily, as there are no generally accepted guidelines regarding the selection of what constitutes a ‘suitable’ value in this scenario. We took a value of 0.5 or greater to represent a cell that was more likely than not to contain at least one site that would act as a suitable den for a spotted-tailed quoll. This output can be seen in supplementary information Fig. 5. We then used the focal statistics tool to calculate for each cell the mean value of all cells within the surrounding 372 hectares, which is the mean home range of a Tasmanian spotted-tailed quoll^37^, and within the surrounding 862 hectares, which is the maximum reported home range of a spotted-tailed quoll^40^. This returned the proportion of cells within the respective home range value that have a fuzzy denning suitability value of greater than 0.5. With no consistent mean home range for mainland spotted-tailed quolls, we selected the mean Tasmanian home range size of 372 hectares^37^ to represent the most likely home range under suitable conditions. We selected the maximum recorded home range of 862 hectares^40^ to represent the largest likely home range where conditions are less favourable, which reflects lower resource availability, and subsequently larger home ranges^81^. To determine what percent of Kangaroo Island consists of home ranges that contain a suitably large proportion of den sites relative to the rest of the home range, we used the reclassify tool to transform each smoothed raster layer into five equal classes of suitability. We then transformed this layer into polygons in order to find the area that each category encompassed. Tables outlining the area of each class for each home range size are shown in supplementary information Tables 7 and 8.

**Fig. 5:**
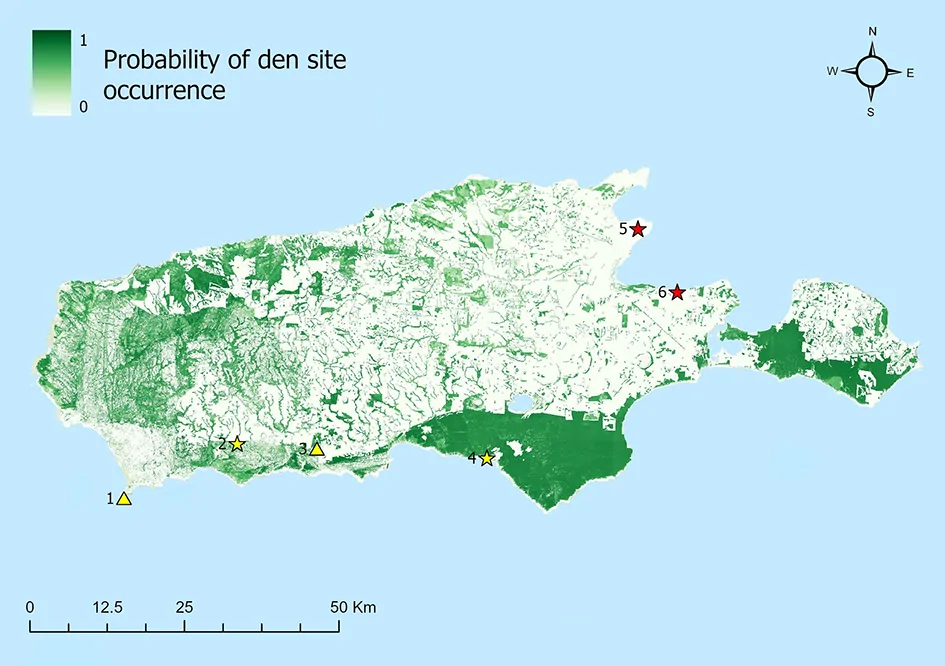
A fuzzy overlay model depicting the likelihood of den site occurrence across Kangaroo Island, where darker green cells are more likely to contain den sites than lighter green cells. Historical records are indicated by the numbered icons, as per Fig. 2. Created using *ArcGIS Pro Version 3.5.1*^55^.

### Code availability

The R code used for the MaxEnt modelling is shown in the supplementary file “MaxEnt_KI.rmd”. For files referenced in R code please see additional supplementary files “Supplementary_table_1-Thinned_occurrences” and “Environmental_data_stack.tif”.

We have detailed the steps required for fuzzy modelling which is shown in supplementary methods. The parameters for fuzzy modelling are shown in supplementary Table 2.

## Results

### Maxent species distribution modelling

MaxEnt modelling (Fig. 3) predicts a habitat suitability score of up to 0.5 on Kangaroo Island. Notably, large portions of their former distribution are predicted to still be suitable. The graph depicting mean area under curve (AUC) (supplementary information Fig. 2) indicates that the model used in assessment, which included linear, quadratic, hinge, product, and threshold (LQHPT) features with a regularisation multiplier (rm) of 0.5, has an AUC of approximately 0.97, showing high test accuracy^82^.

### Fuzzy membership

Fuzzy membership predicts the suitability of Kangaroo Island according to each specific variable. Figure 4 shows the suitability based on clay percentage, mean annual rainfall, understorey density, and foliage cover across Kangaroo Island. Clay percentage along the southern coast of Kangaroo Island stretches between a fuzzy value of zero (> 55% clay) in some sections of the northern half of Kangaroo Island and a fuzzy value of one (< 8% clay) in some locations in the south. Mean annual rainfall has a fuzzy value of one (> 800 mm annual mean rainfall) towards the centre of the western section of Kangaroo Island, and a fuzzy value of zero (< 450 mm) in a few areas of Kangaroo Island, such as in Kingscote. Understory density and foliage cover both have the highest density of fuzzy predicted suitability in Cape Gantheaume, and the southern half of the Dudley Peninsula, and understory density also has high predicted suitability in the western half of the island. The fine resolution of the fuzzy foliage cover layer also allows for fine-scale patterns of foliage cover to be predicted across Kangaroo Island, including along the well-preserved roadside vegetation.

### Fuzzy overlay

The fuzzy overlay model (Fig. 5) predicts fine-scale patterns of habitat suitability across Kangaroo Island. The areas of highest predicted denning suitability are Cape Gantheaume, southern Dudley Peninsula, and large portions of the western end of Kangaroo Island, including high connectivity throughout the island because of high vegetation cover along roadsides and drainage lines. The areas with the lowest density of predicted suitable denning sites are around Kingscote, and suitability in general increases with increasing distance to the west and south, not including the Dudley Peninsula. Approximately 33% of the island has a predicted denning suitability of 0.5 or higher.

### Smoothed models

**Fig. 6:**
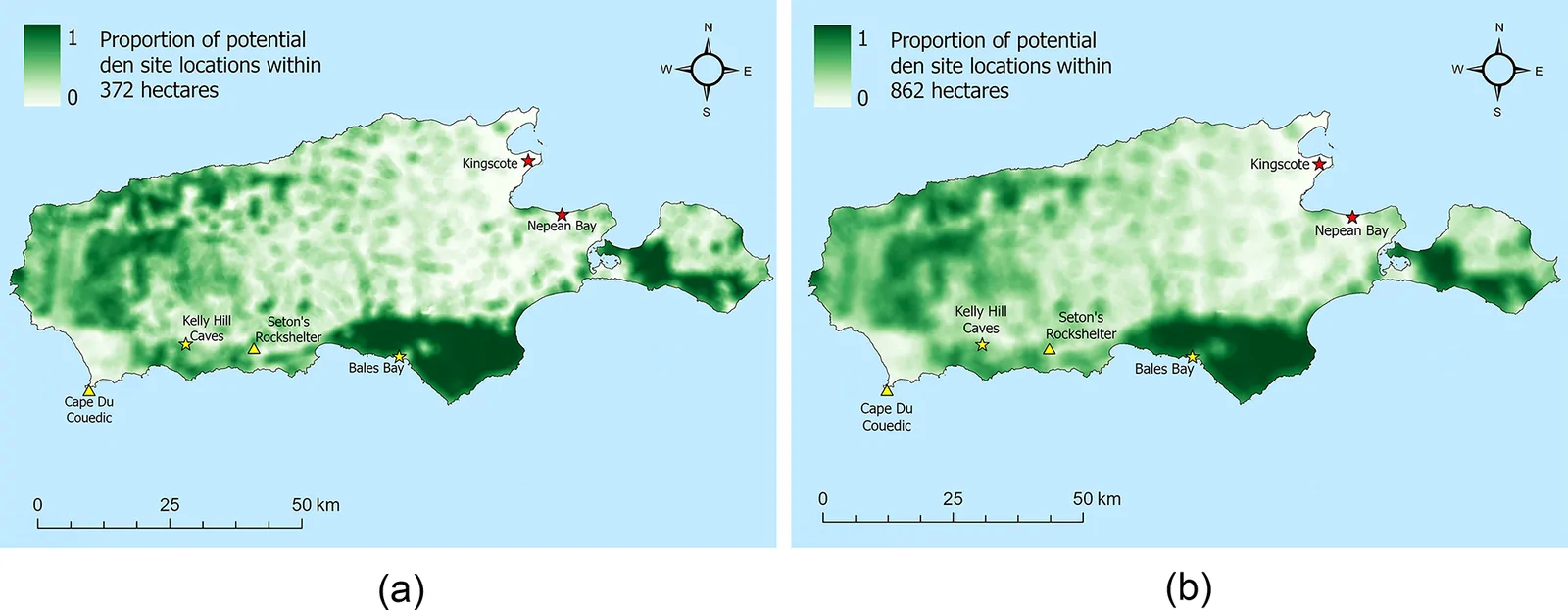
Fuzzy overlay models smoothed to represent the proportion of cells within the maximum known home range of a spotted-tailed quoll (862 hectares; Andrew 2005, left) and the mean home range of a Tasmanian spotted-tailed quoll (372 hectares; Troy 2014, right) that have a denning suitability of 0.5 or greater. Historical records are indicated by the numbered icons, as per Fig. 2. Created using *ArcGIS Pro Version 3.5.1*^55^.

Both smoothed models (Fig. 6) indicate a high density of predicted suitable denning habitat across large portions of Kangaroo Island, with particularly high density in the southern half of the Dudley Peninsula, patches of western Kangaroo Island, and Cape Gantheaume. In both models, the proportion of predicted suitable den sites reaches one at their highest density, meaning that in some locations, every cell within the home range is deemed by the fuzzy model to have a denning suitability of above 0.5, and therefore at least one den site exists within every 10 m cell within the home range. Where a home range of 372 hectares is assumed, 9.3% of Kangaroo Island consists of locations with a proportion of den site availability between 0.8 and 1.0, a further 7.8% falls between a proportion of den site availability of 0.6 and 0.8, 12.2% has a proportion between 0.4 and 0.6, 21.2% has a proportion between 0.2 and 0.4, and 49.4% has a proportion of 0.2 or less. Where a home range of 862 hectares is assumed, 8.2% of Kangaroo Island consists of locations with a proportion of den site availability between 0.8 and 1.0, 7.7% falls within a proportion of den site availability 0.6 and 0.8, 13.3% has a proportion between 0.4 and 0.6, 22.5% has a proportion of between 0.2 and 0.4, and 48.2% has a proportion of less than 0.2. These values are displayed in Table 1.

**Table 1:** The percentage of Kangaroo Island that falls into each den site proportion category when considering home range sizes of 372 ha and 862 ha.

Proportion of den site availability within a home range	Percent of Kangaroo Island with relevant proportion of den site availability when considering a 372 ha home range (%)	Percent of Kangaroo Island with relevant proportion of den site availability when considering a 862 ha home range (%)
0.8–1.0	9.3	8.2
0.6–0.8	7.8	7.7
0.4–0.6	12.2	13.3
0.2–0.4	21.2	22.5
0.0–0.2	49.4	48.2

## Discussion

MaxEnt modelling identified that mean annual rainfall, mean annual temperature (1991–2020), soil clay percentage at 60–100 cm depth, and soil sand percentage at 15–30 cm depth can be used to reliably predict the distribution of spotted-tailed quolls at a broad scale. This broad-scale modelling suggests that Kangaroo Island falls within a suitable range of climatic and edaphic constraints for the species. Fine scale fuzzy modelling revealed distinct patterns of predicted habitat suitability for denning on Kangaroo Island. Estimates of the proportion of available den sites within a home range demonstrated that multiple locations contain high densities of predicted suitable denning locations, including Cape Gantheaume, large parts of western Kangaroo Island and the Dudley Peninsula. This predicted habitat suitability suggests that these locations could be suitable sites for future spotted-tailed quoll reintroductions.

The MaxEnt model revealed that large parts of the species’ former range on the mainland, where the species once existed but now do not, have similar predicted suitability to the species’ current range. As the environmental variables used in our modelling were based on habitat preferences of extant quoll populations, this might, at first glance, suggest that our modelled environmental variables are not reliable for predicting current habitat suitability. However, while these environmental variables might also reliably predict parts of the species’ former range, this may be because the former range approximates the species’ fundamental habitat niche, while the current range reflects the species’ realised niche. The realised niche is a subset of the fundamental niche that is likely constrained by other factors, such as the presence of introduced predators, that are not represented by variables in our MaxEnt model. This is supported in literature, where different habitat requirements have been reported between different spotted-tailed quoll populations, particularly between populations in areas with high fox density compared to those in areas with no or low fox densities, such as Tasmania and the Hunter Valley^36,37,83^. In the literature, spotted-tailed quolls are often referred to as “forest-dependent”, however most of these studies have been conducted in mainland forested habitat in locations with high fox densities. Interactions with introduced predators can potentially force spotted-tailed quolls into smaller parts of their preferred niche, where they can use densely forested habitat to reduce encounters with, or competition from, foxes^37^ (i.e. their realised niche). Hence our MaxEnt modelled habitat suitability may reflect the species’ fundamental habitat niche in the absence of foxes, which is appropriate for the fox-free Kangaroo Island.

Our MaxEnt modelling predicted that Kangaroo Island contains habitat with moderate suitability of up to 0.5. This suitability can be seen as a positive outcome, taking into account that the species is currently absent from much of the region for model training. MaxEnt output represents the relative suitability of landscapes based on the realised niche which may differ from the species’ full potential distribution. Areas with lower predicted values may still be capable of supporting the species, as indicated by occurrence records existing in locations with habitat suitability as low as 0.002. The moderate level of suitability also suggests that occurrence is likely heavily influenced by additional variables that were not included in the MaxEnt model, such as foliage cover and understorey vegetation density (as predictors of den site suitability). Therefore, while higher values suggest increased edaphic and climatic suitability for spotted-tailed quolls, lower suitability values (such as those across Kangaroo Island) do not necessarily represent inhabitable locations. The AUC value of 0.97 clearly demonstrates that the MaxEnt model provides high discriminatory ability and provides meaningful predictions of habitat suitability based on the selected environmental variables at the broad scale. This shows that at a broad scale, Kangaroo Island is within the range of suitable habitat for the spotted-tailed quoll.

At the fine scale required for reintroduction planning, our fuzzy modelling identified distinct patterns of predicted habitat suitability across Kangaroo Island. The key advantage of fuzzy modelling is that it provides a mathematical framework to simulate our reasoning. This is based on a limited number of historical records combined with empirical knowledge provided by experts in the field and ultra high resolution spatial surveying data. The advantage of this is that it provides reproducible estimates of habitat at detail that is relevant for management, which in our case amounted to estimates at 450,000 locations. The disadvantage, however, is that information at such spatial detail cannot be validated.

The smoothed versions of the fuzzy model demonstrate that for large areas of Kangaroo Island, a high proportion of habitat within a spotted-tailed quoll’s home range is likely to contain suitable den sites. Unfortunately, due to the rarity of studies investigating denning requirements of spotted-tailed quolls, the proportion of suitable denning habitat required within a home range is unknown. Various studies have demonstrated that quolls tend to change den sites frequently, often daily, and that they prefer to select their core home ranges in areas with a high density of available den sites. However, the number and density of den sites required are unknown, with differences observed between studies. For example, Glen and Dickman^39^ suggested that individual quolls may use up to nine different dens, changing between them frequently, while Belcher^83^ found individual female quolls utilising > 15 den sites over a period of nine months^83^, except during the breeding season when four to six dens were recorded per individual^69^. Some studies have found that quolls rarely return to the same den^41^, while others report repeated use of the same dens, especially during the breeding season^69^. Studies have shown that core home ranges are more frequently found within forested areas, and that home ranges are generally selected to encompass locations that have multiple den sites clustered together^37,84^. Therefore, areas containing a higher proportion of habitat with a high likelihood of den site availability are more likely to be selected as locations for an individual’s core home range. Regardless of whether a home range of 372 ha or 862 ha is assumed, the highest proportion of available den sites are located in Cape Gantheaume, south and south-western Dudley Peninsula, and the western half of the island, suggesting that these locations would be potential suitable sites for reintroducing spotted-tailed quolls to Kangaroo Island.

Most historic records on Kangaroo Island align with locations deemed suitable by the fuzzy model. The high proportion of den sites predicted at Kelly Hill Caves, Seton’s Rockshelter, and Bales Bay coincide with historical sightings on Kangaroo Island. Additionally, while the exact shoreline where a quoll was sighted by Baudin’s crew is unknown^22^, the section of suitable habitat that our model identified near Nepean Bay (where they are believed to have anchored), may have been associated with the location of the sighting. The Kingscote observational account and the specimen found at Cape du Couedic appear to be in areas which the model has deemed as currently unsuitable. This may reflect changes since European arrival, such as vegetation clearing for development around Kingscote, or it could reflect a gap in the modelling. The habitat around Cape du Couedic is sparsely vegetated, particularly near the coast, but instead is quite rocky with many outcrops. Considering the ability for spotted-tailed quolls to utilise rock shelters^38,39,41^, this may constitute suitable habitat nonetheless. Future studies should consider rockiness as a potential influential variable, if a suitable dataset can be sourced.

Limitations to our model exist, which may have resulted in over- or under-estimation of den site availability in some locations. Not only did this model not consider the use of rock dens by spotted-tailed quolls, but it also did not account for the potential for quolls to den in non-natural structures due to the lack of data quantifying the prevalence of these types of shelters in the landscape. Quolls are known to occupy den sites such as shallow scrapes in the earth, under bridges, and in piles of timber offcuts^39^, demonstrating their ability to make use of a wide variety of non-natural structures as den sites. Furthermore, there was no direct measure of burrowing capability included in this analysis, as there was not enough evidence to suggest that burrows would make up a large portion of den sites in the absence of burrowing species by which dens could be created^40^. Captive quolls have been seen to dig their own burrows, however this behaviour has never been observed in the wild, where burrows occupied by quolls tend to have been constructed by other burrowing species such as wombats and rabbits^40^. The relationship between soil clay percentage and spotted-tailed quoll distribution may reflect soil characteristics affecting burrowing capacity, as soil characteristics are known to influence the capacity for species to construct burrows of appropriate shapes and sizes without risk of collapse^85^. Accordingly, the inclusion of this variable may indirectly account for burrowing ease, but this relationship could also reflect the influence of soil characteristics on vegetation^86^. Imperfect detection, which often occurs due to observation bias towards areas highly frequented by humans^87^, is irrelevant in our model, as the entire Island falls within suitable climatic and edaphic constraints for the species.

A further limitation of the present study is the inability for the fuzzy overlay tool to weight variables, resulting in equally weighted variables in analyses. This is not necessarily representative of the true response of spotted-tailed quolls to each variable, but considering the lack of knowledge regarding these responses, the fuzzy overlay method provides a good approximation of the response of spotted-tailed quolls to the combination of these variables on Kangaroo Island. Furthermore, the model does not provide insight into the dynamic nature of these variables on Kangaroo Island, which is a fundamental flaw of species distribution modelling. The LiDAR derived foliage cover data was collected after the recent fires on Kangaroo Island, so changes to habitat suitability across the island may occur as vegetation continues to recover. Future research may focus on analysis of the effect of vegetation regrowth on the suitability of Kangaroo Island for spotted-tailed quolls, pending the creation of updated LiDAR foliage cover modelling.

## Conclusions

We have successfully implemented a two-tier modelling approach designed to address situations where empirical data for the decision process is limited. At the broad scale, we used empirical species distribution modelling (MaxEnt) to quantify the influence of key climatic variables on habitat suitability, drawing on occurrence records that effectively deals with limited empirical information. At the fine scale, we applied a rule-based fuzzy modelling framework that integrates ecological knowledge of the species denning requirements with high resolution vegetation structure data derived from airborne LIDAR. This combination enables predictions that reflect both the general environmental constraints on the species’ range and the local availability of critical microhabitat.

Results indicate that Kangaroo Island holds considerable potential as a reintroduction site for the spotted-tailed quoll. Broad-scale habitat modelling indicates that the island lies within the species’ preferred broader climatic and soil conditions, with mean annual rainfall, mean annual temperature, and soil texture being the most influential variables. At the finer scale, fuzzy modelling based on high-resolution LIDAR foliage data identified several areas with a high density of potential denning habitat, supporting multiple options for selecting reintroduction sites.

## Supplementary Information

Below is the link to the electronic supplementary material.

Supplementary Material 1

Supplementary Material 2

Supplementary Material 3

Supplementary Material 4

## Data Availability

The species occurrence datasets analysed during this study are available at the Atlas of Living Australia, Dasyurus maculatus: Bindjulang (https://bie.ala.org.au/species/, https://biodiversity.org.au/afd/taxa/617e069f-eb5c-40ec-a027-f5fd40e5145d). The soil Sand (https://data.csiro.au/collection/csiro:10149%3Fq=sand%26%5Fst=keyword%26%5Fstr=34%26%5Fsi=1)and Clay (https://data.csiro.au/collection/csiro%3A55684v5) datasets used in analysis are publicly accessible through the CSIRO data access portal, and mean annual rainfall (https://www.bom.gov.au/climate/maps/averages/rainfall/?period=an®ion=sa) and mean annual temperature (https://www.bom.gov.au/climate/maps/averages/temperature/?maptype=mean&period=an®ion=sa) datasets are publicly available through the Bureau of Meteorology website. The LiDAR dataset used in fuzzy analysis is available by reasonable request from the authors and the Department for Environment and Water.
