# Supplementary material for: Returning an extirpated native carnivore to its former range: a habitat suitability analysis of Kangaroo Island for spotted-tailed quolls (Dasyurus maculatus)
Source: Sci Rep. 2026 Sep 15;16:28733. doi: 10.1038/s41598-026-56577-4 (PMC13578372; doi:10.1038/s41598-026-56577-4)
Supplement: Supplementary file 1 [file 41598_2026_56577_MOESM4_ESM.docx]

## Supplementary information for: “Returning an extirpated native carnivore to its former range: a habitat suitability analysis of Kangaroo Island for spotted-tailed quolls (*Dasyurus maculatus*)”

Authors: Mia Retallack, Bertram Ostendorf, Bronwyn Fancourt, David Peacock

**Supplementary methods:**

**Fuzzy modelling:**

1. We used the fuzzy membership tool in ArcGIS Pro^1^ to reclassify each layer of the fuzzy model such that each cell is assigned a value between zero to one, where values closer to one are more likely to belong to a suitable class, and values closer to zero are less likely to belong to a suitable class. The membership types and parameters used are outlined in supplementary table 7.

Due to the configuration of the NVIS dataset, the fuzzy membership tool cannot be used for reclassification. Instead, we used the NVIS structural formation terminology, as defined in table 7 of the Australia vegetation attribute manual^2^ to define the approximate understorey density of each major vegetation subgroup. This interpretation is outlined in supplementary table 4. We then manually defined the fuzzy ranking for each MVS category in ArcGIS Pro and used the polygon to raster tool to transform this layer based on the fuzzy rankings values.

2. We then used the fuzzy overlay tool in ArcGIS Pro to combine the rasters layers with a resolution of 10 m. The gamma overlay type was used as it returns values which are a combination of all input values, but returns less extreme values than other overlay types^3^. We used the default gamma value of 0.9, which is generally accepted as suitable^4^.

**Supplementary figures:**

**Supplementary figure 1**: A map of Australia depicting the polygon used to delimit the boundary used for MaxEnt modelling (black outline) and the cleaned and thinned (red) and original (blue) spotted-tailed quoll occurrence datasets, including a zoomed map frame to highlight the reduction of survey bias that results from thinning the data to remove spatially adjacent points. Created using [ArcGIS Pro Version 3.5.1](https://www.esri.com/en-us/home)^1^. Background sources Esri, Garmin, FAO, NOAA, USGS.

**Supplementary figure 2**: Average AUC value for each rm value, for each model output. The model chosen (LQHPT, rm = 0.5) has the highest AUC value of approximately 0.97, demonstrating high model accuracy.

**Supplementary figure 3:** Response curve for mean annual rainfall (1991-2020)^5^ returned by the MaxEnt algorithm.

**Supplementary figure 4:** Response curve for soil clay percentage (60-100cm depth)^6^ returned by the MaxEnt algorithm.

**Supplementary figure 5:** A map of Kangaroo island depicting the cells that have a greater than 0.5 probability of den site occurrence in green and those that have a less than 0.5 probability of den site occurrence in white. Created using [ArcGIS Pro Version 3.5.1](https://www.esri.com/en-us/home)^1^.

**Supplementary tables:**

**Supplementary table 1:** A table of occurrence points after the data was thinned and cleaned.

For supplementary table one, see the additional CSV file: “Supplementary_table_1-Thinned_occurrences.csv”

**Supplementary table 2:** Membership types used for fuzzy membership layers.

| **Layer** | **Membership type** | **Parameters** | **Result** |
| --- | --- | --- | --- |
| Mean annual rainfall (1991-2020) | Linear | Minimum: 450mm  Maximum: 800mm | Cells with a value of ≤450mm are assigned a membership value of zero, and cells with a value of ≥800mm are assigned a membership value of one. Values in between are assigned membership values increasing linearly between zero and one. |
| Soil clay percentage (60-100cm depth) | Linear | Minimum: 55%  Maximum: 8% | Cells with ≤ 8% clay content are assigned a value of one, and cells with a ≥ 55% clay content are assigned a value of zero, with a linear decrease in membership between the two values. |
| Foliage cover (%) | Linear | Minimum: 0%  Maximum: 100% | Cells with 0% foliage are assigned a membership value of zero, and cells with 100% foliage cover are assigned a membership value of one, with a linear increase in membership between the two values. |

**Supplementary table 3**: Variables used in the final MaxEnt model, and their sources^5–9^.

| Variable | Source |
| --- | --- |
| Mean annual rainfall 1991-2020 | Bureau of Meteorology (2020a) |
| Mean annual temperature 1991-2020 | Bureau of Meteorology (2020b) |
| Soil clay percentage (60-100cm depth) | CSIRO (Malone & Searle 2022a) |
| Soil sand percentage (15-30cm depth) | CSIRO (Malone & Searle 2022b) |
| Spotted-tailed quoll species occurrence | Atlas of Living Australia (2023) |

**Supplementary table 4**: Variables used in fuzzy modelling, and their sources ^5,6,10,11^.

| Variable | Source |
| --- | --- |
| Mean annual rainfall (1991-2020) | Bureau of Meteorology (2020a) |
| Soil clay percentage (60-100cm depth) | CSIRO (Malone & Searle 2022a) |
| Foliage cover (%) | Department for Environment and Water (2020) |
| Understory density | Department of Climate Change (2020) |

**Supplementary table 5:** The functions of each membership type available for use in the ArcGIS Pro fuzzy membership tool^12^.

| **Variable type** | **Description** |
| --- | --- |
| Fuzzy gaussian | Variables are reclassified into a normal distribution, where the midpoint, specified by the user, is the ideal variable value, and suitability decreases with increased distance from the midpoint. The slope of the transition can be specified by the user. Used when the ideal value is near a specific value |
| Fuzzy near | Similarly, to fuzzy gaussian, fuzzy near is used when the ideal value of a variable is near to a specific value, which is assigned a value of one. Membership decreases with distance from the midpoint. Generally, fuzzy near is used over fuzzy gaussian when the ideal values are more likely to be very close to the midpoint. |
| Fuzzy large | Fuzzy large assigns higher fuzzy membership values to larger values of the input variable. A user-specified midpoint defines the value of the input variable which will have a fuzzy membership of 0.5. The shape of the slope is defined by the spread parameter which can also be altered by the user. |
| Fuzzy MSlarge | Fuzzy MS large is similar to fuzzy large but is more applicable when very large values are more likely to be a member of the set. It is calculated based on the mean and standard deviation of the input variable, and the shape of the slope can be changed by altering the multipliers of the mean and standard deviation. |
| Fuzzy small | Fuzzy small assigns higher fuzzy membership values to smaller values of the input variable. The midpoint is specified by the user and defines the value of the input variable which will have a fuzzy membership of 0.5. The shape of the slope is defined by the spread parameter which can also be altered by the user. |
| Fuzzy MSsmall | Fuzzy MS small is similar to fuzzy small but is more applicable when very small values are more likely to be a member of the set. It is calculated based on the mean and standard deviation of the input variable, and the shape of the slope can be changed by altering the multipliers of the mean and standard deviation. |
| Fuzzy linear | A linear function is applied to reclassify variables. The minimum and maximum variable values are defined by the user, where anything beyond the maximum value is assigned a value of one, and anything beyond the minimum is assigned a value of zero. Values between the minimum and maximum are assigned values of between zero and one in a linear fashion. A negative relationship can be defined by assigning a minimum value that is greater than the maximum value. |

**Supplementary table 6:** The NVIS MVS classifications and the percent understory cover as defined by the NVIS structural formation terminology defined in the Australian Vegetation Attribute Manual^13^.

| **MVS number** | **MVS name** | **Approximate % understorey cover*** | **Fuzzy ranking** |
| --- | --- | --- | --- |
| 4 | Eucalyptus open forests with a shrubby understorey | 50 | 0.5 |
| 5 | Eucalyptus open forests with a grassy understorey | 50 | 0.5 |
| 8 | Eucalyptus woodlands with a shrubby understorey | 50 | 0.5 |
| 12 | Callitris forests and woodlands | 5 | 0.05 |
| 15 | Melaleuca open forests and woodlands | 5 | 0.05 |
| 16 | Other forests and woodlands | 5 | 0.05 |
| 21 | Other Acacia tall open shrublands and [tall] shrublands | 50 | 0.5 |
| 24 | Acacia (+/- low) open woodlands and shrublands +/- tussock grass | 20 | 0.2 |
| 25 | Acacia (+/- low) open woodlands and sparse shrublands with a shrubby understorey | 5 | 0.05 |
| 26 | Casuarina and Allocasuarina forests and woodlands | 5 | 0.05 |
| 28 | Low closed forest or tall closed shrublands (including Acacia, Melaleuca and Banksia) | 85 | 0.85 |
| 29 | Mallee with a dense shrubby understorey | 85 | 0.85 |
| 30 | Heathlands | 50 | 0.5 |
| 31 | Saltbush and bluebush shrublands | 50 | 0.5 |
| 32 | Other shrublands | 50 | 0.5 |
| 36 | Temperate tussock grasslands | 50 | 0.5 |
| 38 | Wet tussock grassland with herbs, sedges or rushes, herblands or ferns | 50 | 0.5 |
| 39 | Mixed chenopod, samphire +/- forbs | 50 | 0.5 |
| 42 | Naturally bare, sand, rock, claypan, mudflat | 0 | 0 |
| 44 | Freshwater, dams, lakes, lagoons or aquatic plants | 0 | 0 |
| 46 | Sea, estuaries (includes seagrass) | 0 | 0 |
| 47 | Eucalyptus open woodlands with shrubby understorey | 50 | 0.5 |
| 48 | Eucalyptus open woodlands with a grassy understorey | 50 | 0.5 |
| 49 | Melaleuca shrublands and open shrublands | 40 | 0.4 |
| 53 | Eucalyptus low open woodlands with a shrubby understorey | 50 | 0.5 |
| 61 | Mallee with a tussock grass understorey | 35 | 0.35 |
| 68 | Open mallee woodlands and sparse mallee shrublands with an open shrubby understorey | 5 | 0.05 |
| 74 | Casuarina and Allocasuarina open woodlands with a shrubby understorey | 50 | 0.5 |
| 75 | Melaleuca open woodlands | 5 | 0.05 |
| 97 | Unclassified native vegetation | 0 | 0 |
| 98 | Cleared, non-native vegetation, buildings | 0 | 0 |
| 99 | Unknown/no data | 0 | 0 |

**Supplementary table 7:** The proportion and percent cover of each class of proportion of den sites within 372 hectares.

| **Proportion of den sites within 372 hectares** | **Area (km^2^)** | **Percent cover of entire island** |
| --- | --- | --- |
| ≤ 0.2 | 2175.86 | 49.4% |
| ≤ 0.4 | 932.36 | 21.2% |
| ≤ 0.6 | 539.53 | 12.2% |
| ≤ 0.8 | 346.65 | 7.8% |
| ≤ 1 | 410.22 | 9.3% |

**Supplementary table 8:** The proportion and percent cover of each class of proportion of den sites within 862 hectares.

| **Proportion of den sites within 862 hectares** | **Area (km^2^)** | **Percent cover of entire island** |
| --- | --- | --- |
| ≤ 0.2 | 2121.14 | 48.2% |
| ≤ 0.4 | 993.08 | 22.5% |
| ≤ 0.6 | 587.04 | 13.3% |
| ≤ 0.8 | 340.75 | 7.7% |
| ≤ 1 | 362.60 | 8.2% |

**References:**

1. ESRI. ArcGIS Pro (version 3.5.1). (2023).
2. NVIS Technical Working Group. Australian Vegetation Attribute Manual V 7.0. (2017)
3. ESRI. How Fuzzy Overlay works. (2023)
4. Gwynn, V. & Symeonakis, E. Rule-based habitat suitability modelling for the reintroduction of the grey wolf (Canis lupus) in Scotland. *PloS One* **17**, e0265293 (2022).
5. Bureau of Meteorology. *High Resolution Mean Monthly, Seasonal and Annual Rainfall Data (Base Climatological Data Sets).* (2020).
6. Malone, B. & Searle, R. *Soil and Landscape Grid National Soil Attribute Maps - Clay (3" Resolution)*. (2022).
7. Bureau of Meteorology. *Mean Monthly and Mean Annual Maximum, Minimum and Mean Temperature Data (Base Climatological Data Sets)*. (2020).
8. Malone, B. & Searle, R. *Soil and Landscape Grid National Soil Attribute Maps - Sand (3" Resolution)*. (2022).
9. Atlas of Living Australia. Dasyurus maculatus (Kerr, 1792). (2023).
10. Department for Environment and Water. *RFQ F0003001501: South Australia LiDAR 2020 - Kangaroo Island*. (2020).
11. Department of Climate Change. *National Vegetation Information System (NVIS) Version 6.0 - AUSTRALIA - Extant Vegetation*. (2020).
12. ESRI. How Fuzzy Membership works. (2023).
13. Australian Vegetation Attribute Manual V 7.0.
